# Supplementary figures and images for: Diversity and pathogenicity of Alternaria species associated with the invasive plant Ageratina adenophora and local plants
Source: PeerJ. 2022 Feb 28;10:e13012. doi: 10.7717/peerj.13012 (PMC8893028; doi:10.7717/peerj.13012)

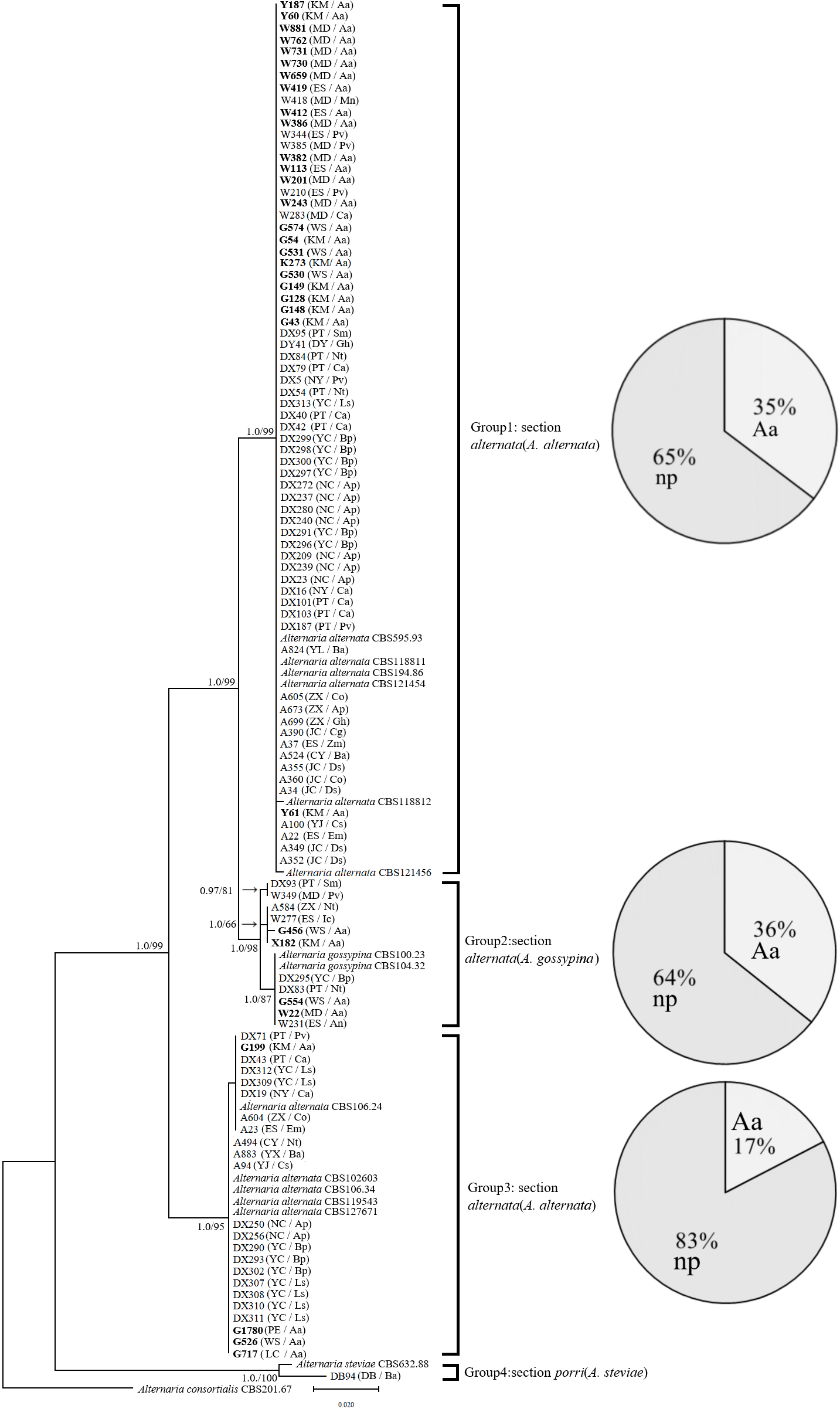

Supplement: Supplemental Information 1 — The numbers above branches represent Bayesian posterior probabilities and maximum-likelihood bootstrap percentages (PP/ML). Only bootstrap percentages over 50% and significant Bayesian posterior probability (0.8) are shown on the branches. The geographic location and plant source for each strain are shown in parentheses following the strain number. The numbers in bold are isolates from A. adenophora. Geographic location: CY-Cangyuan, DB-Debao, DY-Duyun, ES-Eshan, JC-Jianchuan, KM-Kunming, LC-Lancang, MD-Midu, NC-Nanchong, NY-Nayong, PE-Puer, PT-Pingtang, WS-Weishan, YJ-Yuanjiang, YL-Yiliang, YX-Yunxian, ZX-Zhenxiong; plant source: Aa-Ageratina adenophora, An-Alnus nepalensis, Ap-Amygdalus persica, Ba-BetuLa alnoides, Bp-Brassica pekinensis, Ca-Capsicum annuum, Co-Cynanchum otophyllum, Cs-Camellia sinensis, Ds-Dioscorea subcalva, Em-Euphorbia milii, Fm-Fallopia muLtiflora, Gh-Gonostegia hirta, Ic-Imperata cylindrica, Ls-Lactuca sativa, Mn-Musa nana, Nt-Nicotiana tabacum, Pv-Phaseolus vulgaris, Ri-Reinwardtia indica, Sm-Solanum melongena, Zm-Zehneria maysorensis. The tree was rooted to A. consortialis (CBS201.67). The right side of each group shows the percentage of strains isolated from invasive plants (Aa, A. adenophora) and native plants (np, native plant) in this group. [file peerj-10-13012-s001.png]

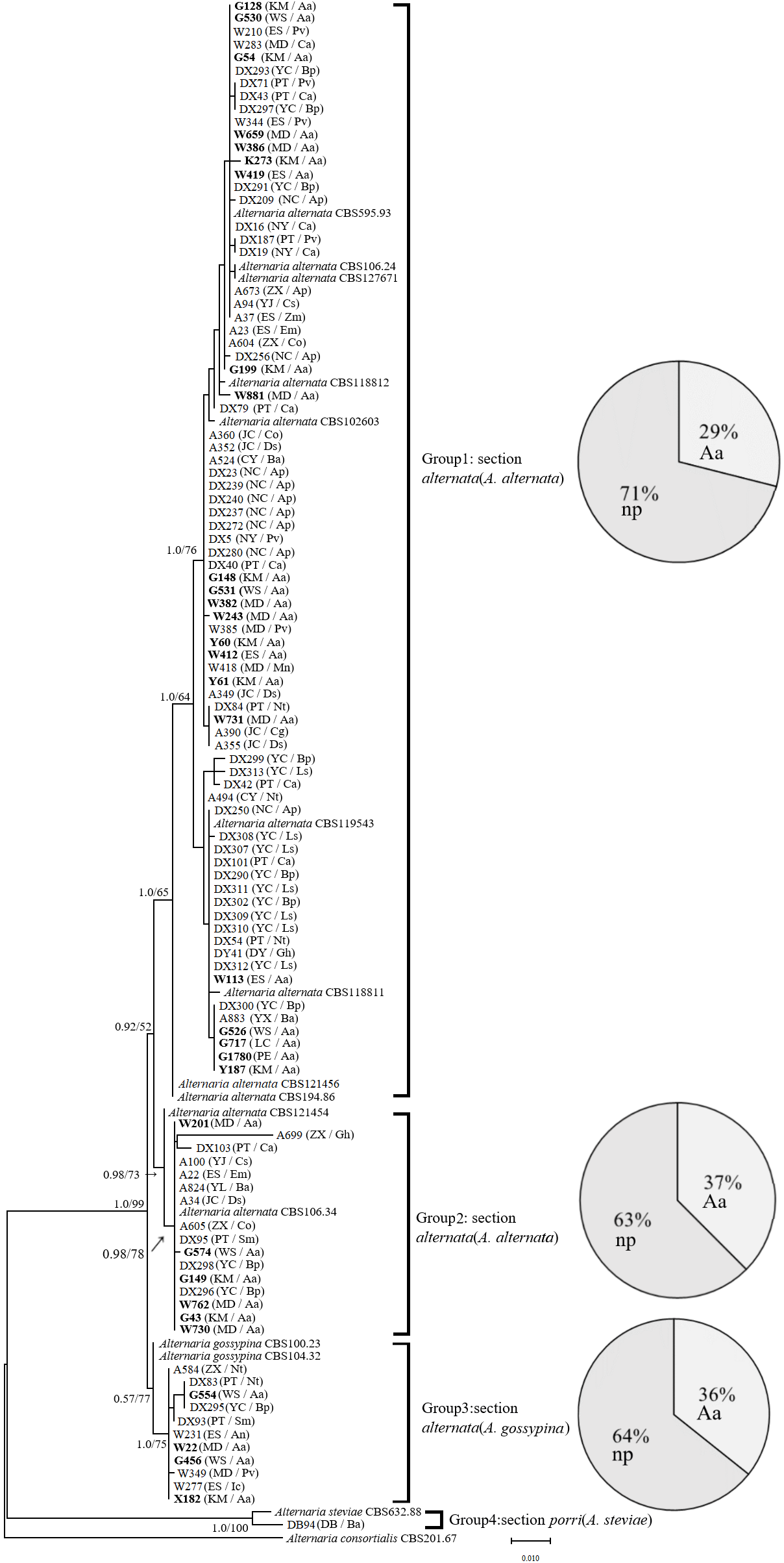

Supplement: Supplemental Information 2 — The numbers above branches represent Bayesian posterior probabilities and maximum-likelihood bootstrap percentages (PP/ML). Only bootstrap percentages over 50% and significant Bayesian posterior probability (0.8) are shown on the branches. The geographic location and plant source for each strain are shown in parentheses following the strain number. The numbers in bold are isolates from A. adenophora. Geographic location: CY-Cangyuan, DB-Debao, DY-Duyun, ES-Eshan, JC-Jianchuan, KM-Kunming, LC-Lancang, MD-Midu, NC-Nanchong, NY-Nayong, PE-Puer, PT-Pingtang, WS-Weishan, YJ-Yuanjiang, YL-Yiliang, YX-Yunxian, ZX-Zhenxiong; plant source: Aa-Ageratina adenophora, An-Alnus nepalensis, Ap-Amygdalus persica, Ba-BetuLa alnoides, Bp-Brassica pekinensis, Ca-Capsicum annuum, Co-Cynanchum otophyllum, Cs-Camellia sinensis, Ds-Dioscorea subcalva, Em-Euphorbia milii, Fm-Fallopia muLtiflora, Gh-Gonostegia hirta, Ic-Imperata cylindrica, Ls-Lactuca sativa, Mn-Musa nana, Nt-Nicotiana tabacum, Pv-Phaseolus vulgaris, Ri-Reinwardtia indica, Sm-Solanum melongena, Zm-Zehneria maysorensis. The tree was rooted to A. consortialis (CBS201.67). The right side of each group shows the percentage of strains isolated from invasive plants (Aa, A. adenophora) and native plants (np, native plant) in this group. [file peerj-10-13012-s002.png]

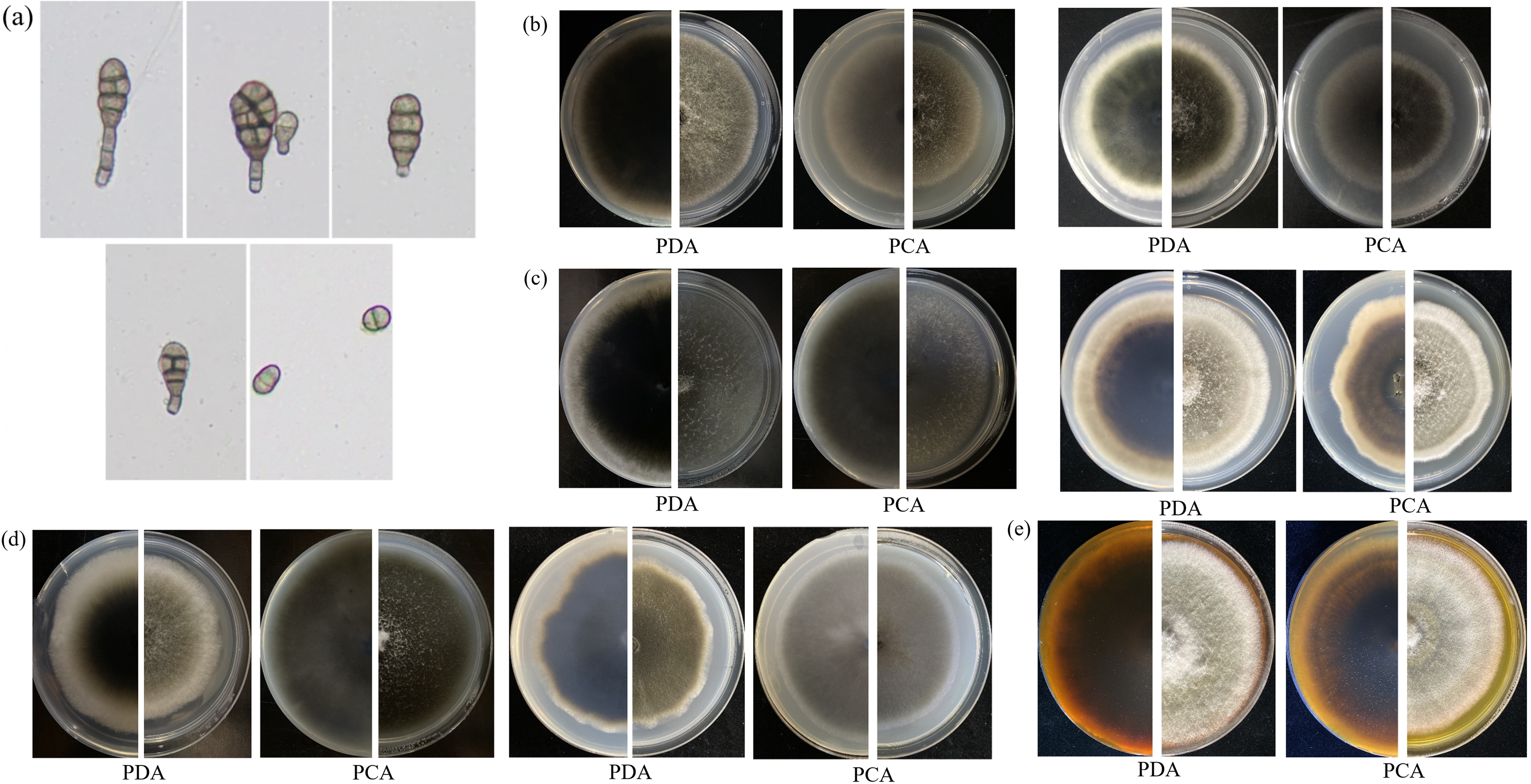

Supplement: Supplemental Information 3 — (a) conidia; (b) Colony morphology of the genotype A. alternata (group1) on PDA and PCA; (c) Colony morphology of the genotype A. gossypina (group2) on PDA and PCA; (d) Colony morphology of the genotype A. alternata (group3) on PDA and PCA. On the left is the colony morphology of most of the isolates in each region, Others are the colony morphology of individual isolates; (e) Colony morphology of the genotype A. steviae (group4) on PDA and PCA, and it produced orange pigment. [file peerj-10-13012-s003.png]
