# Supplementary material for: Diversity and pathogenicity of Alternaria species associated with the invasive plant Ageratina adenophora and local plants
Source: PeerJ. 2022 Feb 28;10:e13012. doi: 10.7717/peerj.13012 (PMC8893028; doi:10.7717/peerj.13012)
Supplement: Supplemental Information 4 [file peerj-10-13012-s004.docx]

| Locality | Chinese province | Longitude (E) | Latitude (N) | Isolates from *A. adenophora* | Isolates from native plants |
| --- | --- | --- | --- | --- | --- |
| Kunming | Yunnan | 102.63 | 24.85 | 11 | 0 |
| Cangyuan | Yunnan | 99.34 | 23.34 | 0 | 2 (*BetuLa alnoides*, *Nicotiana tabacum*) |
| Lancang | Yunnan | 99.71 | 22.76 | 1 | 0 |
| Puer | Yunnan | 100.81 | 22.75 | 1 | 0 |
| Yunxian | Yunnan | 100.23 | 24.66 | 0 | 1 (*BetuLa alnoides*) |
| Yuanjiang | Yunnan | 102.00 | 23.56 | 0 | 2 (*Camellia sinensis*) |
| Yiliang | Yunnan | 104.06 | 27.61 | 0 | 1 (*BetuLa alnoides*) |
| Zhenxiong | Yunnan | 104.86 | 27.42 | 0 | 5 (*Amygdalus persica*, *Nicotiana tabacum*, *Cynanchum otophyllum* Schneid, *Gonostegia hirta*) |
| Jianchuan | Yunnan | 100.21 | 24.46 | 0 | 6 (*Dioscorea subcalva*, *Cynanchum otophyllum*, *Cyclobalanopsis glaucoides*, *Zehneria maysorensis*) |
| Weishan | Yunnan | 100.35 | 25.13 | 6 | 0 |
| Midu | Yunnan | 100.49 | 25.37 | 10 | 4 (*Phaseolus vulgaris*, *Musa nana*, *Capsicum annuum*) |
| Eshan | Yunnan | 102.29 | 24.12 | 3 | 7 (*Phaseolus vulgaris*, *Euphorbia milii*, *Alnus nepalensis*, *Zehneria maysorensis*, *Imperata cylindrica*) |
| Nayong | Guizhou | 105.58 | 26.83 | 0 | 4 (*Capsicum annuum*, *Phaseolus vulgaris*m, *Amygdalus persica*) |
| Pingtang | Guizhou | 107.06 | 25.84 | 0 | 13 (*Nicotiana tabacum*, *Capsicum annuum*, *Solanum melongena*, *Phaseolus vulgaris*) |
| Duyun | Guizhou | 107.51 | 26.25 | 0 | 1 (*Gonostegia hirta*) |
| Nanchong | Sichuan | 106.02 | 30.45 | 0 | 8 (*Amygdalus persica*) |
| Yichang | Hubei | 111.32 | 30.77 | 0 | 17 (*Brassica pekinensis*, *Lactuca sativa*) |
| Debao | Guangxi | 106.57 | 23.34 | 0 | 1 (*BetuLa alnoides*) |

**Table S1 Description of sampling sites and fungal isolation sources**
