# Supplementary material for: Diversity and pathogenicity of Alternaria species associated with the invasive plant Ageratina adenophora and local plants
Source: PeerJ. 2022 Feb 28;10:e13012. doi: 10.7717/peerj.13012 (PMC8893028; doi:10.7717/peerj.13012)
Supplement: Supplemental Information 5 [file peerj-10-13012-s005.docx]

**Table S2 *Alternaria* strains distributed in each groups on different phylogenetic trees**

| Phylogenetic tree | Group | | | | |
| --- | --- | --- | --- | --- | --- |
|  | *A. alternate* | *A. alternate* | | *A. gossypina* | *A. steviae* |
| Alt a1/Alt a1 and Calmodulin | A100 A22 A34 A349 A352 A355 A360 A37 A390 A524 A605 A673 A699 A824 DX101 DX103 DX16 DX187 DX209 DX23 DX237 DX239 DX240 DX272 DX280 DX291 DX296 DX297 DX298 DX299 DX300 DX313 DX40 DX42 DX5 DX54 DX79 DX84 DX95 DY41 G128 G148 G149 G43 G530 G531 G54 G574 K273 W113 W201 W210 W243 W283 W344 W382 W385 W386 W412 W418 W419 W659 W730 W731 W762 W881 Y187 Y60 Y61 (69 isolates) | | A23 A494 A604 A883 A94 DX19 DX250 DX256 DX290 DX293 DX302 DX307 DX308 DX309 DX310 DX311 DX312 DX43 DX71 G1780 G199 G526 G717  (23 isolates) | A584 DX295 DX83 DX93 G456 G554 W22 W231 W277 W349 X182  (11 isolates) | DB94 |
| Calmodulin | A23 A349 A352 A355 A360 A37 A390 A494 A524 A604 A673 A883 A94 DX101 DX16 DX187 DX19 DX209 DX23 DX237 DX239 DX240 DX250 DX256 DX272 DX280 DX290 DX291 DX293 DX297 DX299 DX300 DX302 DX307 DX308 DX309 DX310 DX311 DX312 DX313 DX40 DX42 DX43 DX5 DX54 DX71 DX79 DX84 DY41 G128 G148 G1780 G199 G526 G530 G531 G54 G717 K273 W113 W210 W243 W283 W344 W382 W385 W386 W412 W418 W419 W659 W731 W881 Y187 Y60 Y61 (76 isolates) | A100 A22 A34 A605 A699 A824 DX103 DX296 DX298 DX95 G149 G43 G574 W201 W730 W762  (16 isolates) | | Same as Alt a1 | Same as Alt a1 |
