# Supplementary material for: Diversity and pathogenicity of Alternaria species associated with the invasive plant Ageratina adenophora and local plants
Source: PeerJ. 2022 Feb 28;10:e13012. doi: 10.7717/peerj.13012 (PMC8893028; doi:10.7717/peerj.13012)
Supplement: Supplemental Information 6 — a A100-Y61, A23-G717: Alternaria alternate, A584-X182: Alternaria gossypina, DX94: Alternaria steviae; size number is the average of five repeats of leaf. b Aa, A. adenophora, Ht, Hypoestes trifloral, Ah, Arthraxon hispidu, Ul, Urena lobata, Ab, Ampelopsis bodinieri, Ap, Argyreia pierreana, Fm, Fallopia multiflora, Ct, Celtis tetrandra, Cg, Cyclobalanopsis glaucoides, Lc, Lindera communis [file peerj-10-13012-s006.docx]

| Strain number^a^ | Average spot size / cm^2 b^ | | | | | | | | | |
| --- | --- | --- | --- | --- | --- | --- | --- | --- | --- | --- |
|  | Aa | Ht | Ah | Ul | Ab | Fm | Ap | Ct | Cg | Lc |
| A100 | 0.00 | 0.34 | 0.00 | 0.00 | 0.06 | 0.66 | 0.00 | 0.00 | 0.00 | 0.00 |
| A699 | 0.18 | 0.13 | 0.06 | 0.00 | 0.00 | 0.35 | 0.00 | 0.00 | 0.00 | 0.00 |
| DX101 | 0.35 | 0.28 | 0.07 | 0.07 | 0.00 | 0.12 | 0.08 | 0.00 | 0.00 | 0.00 |
| DX23 | 0.00 | 0.32 | 0.10 | 0.07 | 0.23 | 0.33 | 0.08 | 0.00 | 0.00 | 0.00 |
| DX291 | 0.56 | 0.34 | 0.11 | 0.05 | 0.12 | 0.46 | 0.12 | 0.00 | 0.00 | 0.00 |
| DX296 | 0.53 | 0.10 | 0.00 | 0.00 | 0.47 | 0.99 | 0.22 | 0.07 | 0.00 | 0.04 |
| DX297 | 0.21 | 0.14 | 0.16 | 0.19 | 0.43 | 0.25 | 0.01 | 0.10 | 0.07 | 0.00 |
| DX300 | 0.37 | 0.45 | 0.11 | 0.00 | 0.12 | 0.10 | 0.00 | 0.00 | 0.00 | 0.00 |
| DX313 | 0.14 | 0.02 | 0.00 | 0.00 | 0.20 | 0.45 | 0.09 | 0.00 | 0.00 | 0.00 |
| DX42 | 0.52 | 0.00 | 0.07 | 0.03 | 0.06 | 0.26 | 0.00 | 0.09 | 0.05 | 0.03 |
| DX5 | 0.00 | 0.07 | 0.00 | 0.00 | 0.00 | 0.06 | 0.00 | 0.00 | 0.00 | 0.00 |
| DX54 | 0.37 | 0.08 | 0.00 | 0.00 | 0.00 | 0.04 | 0.00 | 0.00 | 0.00 | 0.02 |
| DX79 | 0.90 | 0.35 | 0.12 | 0.10 | 0.12 | 0.46 | 0.19 | 0.08 | 0.01 | 0.00 |
| DY41 | 0.16 | 0.65 | 0.00 | 0.19 | 0.22 | 0.45 | 0.11 | 0.00 | 0.00 | 0.00 |
| G149 | 0.47 | 0.06 | 0.12 | 0.03 | 0.17 | 2.38 | 0.24 | 0.30 | 0.11 | 0.20 |
| G54 | 0.00 | 0.25 | 0.11 | 0.00 | 0.20 | 0.09 | 0.06 | 0.10 | 0.00 | 0.00 |
| G574 | 0.44 | 0.19 | 0.00 | 0.00 | 0.42 | 0.79 | 0.16 | 0.00 | 0.00 | 0.00 |
| W210 | 0.30 | 0.56 | 0.08 | 0.15 | 0.22 | 0.54 | 0.12 | 0.10 | 0.06 | 0.04 |
| W243 | 0.22 | 0.37 | 0.17 | 0.10 | 0.12 | 0.32 | 0.00 | 0.00 | 0.00 | 0.00 |
| W283 | 0.00 | 0.07 | 0.00 | 0.00 | 0.00 | 0.00 | 0.00 | 0.00 | 0.00 | 0.00 |
| W386 | 0.18 | 0.03 | 0.07 | 0.11 | 0.11 | 0.21 | 0.05 | 0.00 | 0.00 | 0.00 |
| W419 | 0.00 | 0.07 | 0.00 | 0.00 | 0.00 | 0.00 | 0.00 | 0.00 | 0.00 | 0.00 |
| W659 | 0.16 | 0.24 | 0.00 | 0.00 | 0.18 | 0.31 | 0.00 | 0.00 | 0.00 | 0.00 |
| W730 | 0.39 | 0.20 | 0.11 | 0.00 | 0.21 | 0.28 | 0.20 | 0.00 | 0.00 | 0.62 |
| W731 | 0.26 | 0.35 | 0.02 | 0.09 | 0.28 | 0.67 | 0.00 | 0.11 | 0.00 | 0.00 |
| W762 | 0.70 | 0.60 | 0.18 | 0.10 | 0.21 | 0.48 | 0.12 | 0.08 | 0.00 | 0.04 |
| Y187 | 0.81 | 0.56 | 0.00 | 0.13 | 0.87 | 0.37 | 0.07 | 0.02 | 0.00 | 0.00 |
| Y60 | 0.46 | 0.15 | 0.00 | 0.21 | 0.84 | 0.84 | 0.06 | 0.14 | 0.15 | 0.04 |
| Y61 | 0.32 | 0.14 | 0.25 | 0.19 | 0.32 | 1.35 | 0.25 | 0.24 | 0.20 | 0.26 |
| A584 | 0.46 | 0.76 | 0.12 | 0.00 | 0.10 | 0.23 | 0.12 | 0.09 | 0.02 | 0.00 |
| DX295 | 0.33 | 0.36 | 0.11 | 0.13 | 0.57 | 0.29 | 0.02 | 0.08 | 0.02 | 0.04 |
| DX83 | 0.20 | 0.20 | 0.00 | 0.00 | 0.00 | 0.08 | 0.00 | 0.00 | 0.00 | 0.00 |
| DX93 | 0.00 | 0.15 | 0.00 | 0.00 | 0.00 | 0.03 | 0.00 | 0.00 | 0.00 | 0.00 |
| G554 | 0.27 | 0.42 | 0.00 | 0.00 | 0.20 | 0.24 | 0.19 | 0.00 | 0.05 | 0.00 |
| W22 | 0.00 | 0.16 | 0.00 | 0.00 | 0.00 | 0.19 | 0.00 | 0.00 | 0.00 | 0.00 |
| W277 | 0.00 | 0.04 | 0.00 | 0.00 | 0.00 | 0.00 | 0.00 | 0.00 | 0.00 | 0.00 |
| W349 | 0.00 | 0.13 | 0.00 | 0.00 | 0.11 | 0.66 | 0.00 | 0.00 | 0.00 | 0.00 |
| X182 | 0.00 | 0.12 | 0.00 | 0.00 | 0.00 | 0.07 | 0.00 | 0.00 | 0.00 | 0.00 |

**Table S3 Average leaf spots' area after infection by pathogenicity test**

Continue

| A23 | 0.12 | 0.09 | 0.00 | 0.00 | 0.00 | 0.00 | 0.00 | 0.00 | 0.00 | 0.00 |
| --- | --- | --- | --- | --- | --- | --- | --- | --- | --- | --- |
| A494 | 0.57 | 0.56 | 0.38 | 0.02 | 0.17 | 0.27 | 0.24 | 0.00 | 0.02 | 0.00 |
| A94 | 0.45 | 0.12 | 0.23 | 0.12 | 0.29 | 0.98 | 0.02 | 0.12 | 0.02 | 0.00 |
| DX19 | 0.00 | 0.20 | 0.00 | 0.00 | 0.17 | 0.11 | 0.00 | 0.00 | 0.00 | 0.00 |
| DX250 | 0.51 | 0.10 | 0.12 | 0.00 | 0.23 | 0.78 | 0.19 | 0.00 | 0.03 | 0.00 |
| DX256 | 0.62 | 0.46 | 0.00 | 0.00 | 0.18 | 0.55 | 0.00 | 0.00 | 0.00 | 0.04 |
| DX302 | 0.45 | 0.67 | 0.13 | 0.02 | 0.12 | 0.98 | 0.12 | 0.00 | 0.02 | 0.00 |
| DX307 | 0.30 | 0.12 | 0.00 | 0.00 | 0.10 | 0.32 | 0.00 | 0.00 | 0.00 | 0.00 |
| DX308 | 0.55 | 0.06 | 0.00 | 0.00 | 0.22 | 0.78 | 0.15 | 0.07 | 0.00 | 0.04 |
| DX311 | 0.89 | 0.06 | 0.12 | 0.07 | 0.13 | 0.87 | 0.08 | 0.00 | 0.00 | 0.00 |
| DX71 | 0.23 | 0.12 | 0.12 | 0.03 | 0.35 | 0.02 | 0.00 | 0.02 | 0.00 | 0.00 |
| G1780 | 0.09 | 0.51 | 0.05 | 0.04 | 0.62 | 3.32 | 0.57 | 0.08 | 0.05 | 0.32 |
| G717 | 0.25 | 0.26 | 0.23 | 0.04 | 0.25 | 0.25 | 0.00 | 0.10 | 0.00 | 0.00 |
| DB94 | 0.41 | 0.20 | 0.00 | 0.00 | 0.08 | 0.97 | 0.22 | 0.14 | 0.00 | 0.01 |

^a^ A100-Y61, A23-G717: *Alternaria alternate*, A584-X182: *Alternaria gossypina,* DX94: *Alternaria steviae;* size number is the average of 5 repeats of leaf.

^b^ Aa, *A. adenophora*, Ht, *Hypoestes trifloral*, Ah, *Arthraxon hispidu*, Ul, *Urena lobata*, Ab, *Ampelopsis bodinieri*, Ap, *Argyreia pierreana*, Fm, *Fallopia multiflora*, Ct, *Celtis tetrandra*, Cg, *Cyclobalanopsis glaucoides*, Lc, *Lindera communis*
